# Varied flushing frequency and volume to prevent peripheral intravenous catheter failure: a pilot, factorial randomised controlled trial in adult medical-surgical hospital patients

**DOI:** 10.1186/s13063-016-1470-6

**Published:** 2016-07-26

**Authors:** Samantha Keogh, Julie Flynn, Nicole Marsh, Gabor Mihala, Karen Davies, Claire Rickard

**Affiliations:** 1 NHMRC Centre of Research Excellence in Nursing (NCREN), Menzies Health Institute Queensland, Griffith University, Health Sciences Building N48 170 Kessels Road, Nathan, Brisbane, Queensland 4011 Australia; 2 Centre for Clinical Nursing, Royal Brisbane and Women’s Hospital, Herston, Brisbane, Queensland Australia; 3 School of Medicine, Menzies Health Institute Queensland, Griffith University, Meadowbrook, Queensland Australia; 4 School of Medicine, University of Queensland, St Lucia, Brisbane, Australia

**Keywords:** Flushing, 0.9 % sodium chloride, Peripheral, Randomised controlled trial, Catheter obstruction, Vascular access devices, Phlebitis

## Abstract

**Background:**

Research has identified high failure rates of peripheral intravenous catheter (PIVC) and varied flushing practices.

**Methods:**

This is a single-centre, pilot, non-masked, factorial randomised controlled trial. Participants were adults, with a PIVC of expected use ≥24 hours (*n* = 160), admitted to general medical or surgical wards of a tertiary referral hospital in Queensland (Australia). Patients were randomly allocated to one of four flush groups using manually prepared syringes and 0.9 % sodium chloride: 10 mL or 3 mL flush, every 24 or 6 hours. The primary endpoint was PIVC failure, a composite measure of occlusion, infiltration, accidental dislodgement and phlebitis.

**Results:**

PIVC average dwell was 3.1 days. PIVC failure rates per 1000 hours were not significantly different for the volume intervention (4.84 [3 mL] versus 7.44 [10 mL], *p* = 0.06, log-rank). PIVC failure rates per 1000 hours were also not significantly different for the frequency intervention (5.06 [24 hour] versus 7.34 [6 hour], *p* = 0.05, log-rank). Cox proportional hazard regression found neither the flushing nor frequency intervention, or their interaction (*p* = 0.21) to be significantly associated with PIVC failure. However, female gender (hazard ratio [HR] 2.2 [1.3–3.6], *p* < 0.01), insertion in hand/posterior wrist (HR 1.7 [1.0–2.7], *p* < 0.05) and the rate per day of PIVC access (combined flushes and medication pushes) (HR 1.2 [1.1–1.4], *p* < 0.01) significantly predicted PIVC failure.

**Conclusion:**

Neither increased flushing volume nor frequency significantly altered the risk of PIVC failure. Female gender, hand/posterior wrist placement and episodes of access (flushes and medication) may be more important. Larger, definitive trials are feasible and required.

**Trial registration:**

Australian and New Zealand Clinical Trials Registry: ACTRN12615000025538. Registered on 19 January 2015.

## Background

Peripheral intravenous catheters (PIVCs) are the most commonly used invasive devices in hospitals; they are relied upon for therapy across nearly all medical and surgical specialties [1–3]. Yet failure prior to completion of therapy occurs in up to 69 % of patients [4–12]. This may be due to a range of complications, which can be mechanical, vascular or infectious. Mechanical complications include occlusion, infiltration and dislodgement. Vascular complications include venous thrombotic occlusion and phlebitis (irritation or inflammation of the vessel wall). Infectious complications may be bacterial or fungal, and local or systemic bloodstream infections. Complications lead to device failure and device replacement, which results in interrupted therapy, pain associated with resiting and increased health care costs for resources and staff time [13]. Bloodstream infections prolong hospitalisation and increase treatment costs and mortality [14, 15].

The interactions believed to cause mechanical and vascular complications are based on the following theoretical understandings. A fibrin coating forms within the PIVC lumen and catheter tip within 24 hours of placement. Fibrin can form the basis for thrombus development, which as well as being a nidus for infection, can occlude the PIVC lumen and even the vessel [16]. Similarly, inappropriate concentrations of injected/infused solutions, or incompatible mixtures, can cause fluids or medications to precipitate within the catheter lumen and may obstruct the catheter [17].

Current practice recommendations are to flush PIVCs before and after each medication administration, and at regular intervals when PIVCs are not in use [15, 18, 19]. The theoretical purpose of flushing is to maintain catheter patency by preventing internal luminal occlusion, reducing build-up of blood and other products on the PIVC internal surface and preventing interaction of incompatible fluids/medicines [18, 19].

Flushing is a historical practice, based more on derived scientific principles and tradition than on randomised controlled trials (RCTs), and current flushing practices vary widely [20, 21]. This has implications for costs and workload [13]. A large survey of flushing practices revealed a high level of policy awareness (72 %) but varied levels of adherence. Approximately half of respondents stated that there was no medical order or documentation for the flush. Twenty-five percent of respondents used a syringe smaller than the required 10 mL. Use of prefilled syringes was limited to 10 %. The frequency of flushing varied widely from 4th hourly to never, with the most common responses being *pro re nata* (23 %) or 6th hourly (23 %) [20].

There are no RCTs in adult patients comparing different flushing volumes and frequencies that are in common practice today. A single-site paediatric RCT found no significant difference in overall PIVC failure with varied flushing frequencies [22]. Large RCTs in varied patient populations are urgently needed to identify the ideal flushing methods including frequency and volume. This would inform practice to [1–3] prevent PIVC failure, thus preventing painful complications and reinsertions, and reduce organisational costs [13]. This pilot study aimed to test the effect of both volume and frequency on PIVC failure, in preparation for future large trials.

## Methods

The study was a four-arm, 2 × 2 factorial design RCT comparing the effectiveness of different flushing frequencies (more versus less) and volumes (high versus low) in maintaining the patency of PIVCs. A factorial design enables more than one clinical question to be tested from a single RCT [23]. In addition to testing the feasibility of conducting a larger trial, our hypotheses were that both increased flushing volume and frequency would decrease PIVC failure.

### Study interventions

Patients were allocated to one of the four following study arms:

1. High volume, high frequency (10 mL every 6 hours)
2. High volume, low frequency (10 mL every 24 hours)
3. Low volume, high frequency (3 mL every 6 hours)
4. Low volume, low frequency (3 mL every 24 hours)

The volume and frequency parameters were derived from reported practice in a large cross-sectional survey, guidelines and literature [20]. Flushes were 0.9 % sodium chloride using syringes that were manually prepared by ward nursing staff (prefilled syringes were not used). Colour coded stickers indicating the flush prescription were placed in the regular order section of the patient’s medication chart. This order was in addition to the recommended practice of 5–10 mL pre- and post-medication flushes with 0.9 % sodium chloride. Confirmation of intravenous (IV) flush as per protocol was by the nursing signature against prescription in medication chart.

### Insertion and care of PIVCs

Forty-five percent of all PIVCs were inserted by specialist nursing IV inserters. The remainder were inserted by clinical nursing or medical staff. The skin preparation used was chlorhexidine 2 % with isopropyl alcohol 70 % swabs (SOLU-IV^MC/TM^ 3 M St Paul, MN, USA). BD Insyte™ Autoguard™ BC catheters were used with BD Connecta™ extension tubing and CareFusion SmartSite® needleless connectors (BD Medical, Franklin Lakes, NJ, USA). PIVCs were secured with standard simple polyurethane dressings. Nursing (not the research or IV team) and medical staff provided follow-up care. Catheter sites were labelled to identify study inclusion. PIVCs were replaced on clinical indication as per hospital policy, although occasionally some medical staff still requested routine replacement. The Research Nurses (RNs) visited patients daily to visually inspect the PIVC, remind staff of research protocol, gather data collection sheets (until two days after removal of the PIVC) and assess for outcome measures and adverse events. Other data were obtained by the RNs from patient charts, notes, computerised administration and pathology systems.

### Outcome measures

This pilot study collected outcomes to establish feasibility of the protocol and processes. Feasibility data outcomes included the success of screening and recruitment strategies; ease of data collection processes and technology; and resources and research staff time.

The primary endpoint for both the volume and frequency hypotheses was PIVC failure, a composite of any unplanned PIVC removal prior to completion of therapy. This included: occlusion (PIVC will not infuse, or leakage occurs when fluid infused), infiltration (leakage of fluid into surrounding tissues), accidental dislodgement and phlebitis (score of 2 or more of pain/tenderness, redness, swelling, purulence and/or a palpable cord). A secondary outcome was infection (laboratory-confirmed local or bloodstream infection) [24, 25].

### Setting and sample

The trial was undertaken in surgical and medical wards at a large tertiary metropolitan hospital in Brisbane, Australia, where 160 patients were recruited over four months. This number is adequate in pilot trials to represent the target population of larger RCTs for the purposes of assessing feasibility [26].

Potentially eligible patients were those aged 18 years or over, with a PIVC that had been inserted within 24 hours of recruitment, or was about to be inserted and expected to remain for >24 hours, and who gave written informed consent. Exclusion criteria included: non-English-speaking patients without an interpreter and patients receiving continuous IV therapy. Only one PIVC per patient was studied. The study received ethics committee approval from the Royal Brisbane and Women’s Hospital Human Research Ethics Committee (HREC/13/QRBW/420) and Griffith University Human Research Ethics Committee (NRS/12/14/HREC), and the trial was registered with the Australian and New Zealand Clinical Trials Registry: ACTRN12615000025538.

### Recruitment, randomisation, allocation concealment and blinding

Staff education about study aims and processes was conducted prior to commencement of study on all wards. An RN screened patients daily, gained informed consent, performed randomisation and liaised with the ward nursing and medical teams. Eligible patients were invited to participate in the study and written informed consent gained. Randomisation (simple) was obtained via a centralised web-based service (https://www151.griffith.edu.au) and was generated in a 1:1:1:1 ratio for the four study groups. Allocation concealment was maintained until each patient’s study entry. A Project Manager undertook regular quality checks to ensure allocation integrity and data quality. Intravenous flush orders were not amenable to blinding of patients, clinical staff or RNs.

### Data collection

The RNs collected and entered data in the clinical areas using preformatted case report forms and then entered the data into a purpose-built computer database (Research Electronic Data Capture, Vanderbilt University). All data were de-identified at this point and only identifiable within the database by specific study number. Patient characteristics collected by RNs at baseline included: age, sex, estimated weight category, diagnostic group, dominant limb, vein quality, skin integrity, co-morbidities, length of stay, immunodeficiency, current infection and intravenous therapy (including antimicrobial). PIVC characteristics collected were: device type, insertion site, PIVC gauge, side of insertion, clinical area/ward, inserter discipline, initial/subsequent PIVC and the addition of extension tubing and injection ports. The RNs visually inspected PIVCs daily and assessed for phlebitis. After PIVC removal, the following data were collected: reason for PIVC removal, dwell time, phlebitis with 48 hours of removal, hospital length of stay and hospital mortality. The number of PIVC accesses to administer medication/flushes was recorded from the patient’s medication charts. In the case of any suspected blood stream infections, clinical staff ordered blood and PIVC cultures from patients suspected as per usual practice and RNs accessed the results.

### Data analysis

Data cleaning and analyses were performed in Stata (14.1, StataCorp, College Station, TX, USA). An intention-to-treat analysis framework was used. Mean values and standard deviations were reported for normally distributed data; median values and interquartile range were reported otherwise. As a pilot study, we tested our statistical comparison methods, but did not expect to find statistical differences. Incidence rates of PIVC failure per 1000 PIVC hours (95 % confidence intervals [CIs] were calculated, and Kaplan-Meier curves generated. The null hypotheses of no differences in PIVC survival with increased volume or increased frequency were tested with the log-rank test of equality. Cox proportional hazard models (univariable and multivariable) were fitted and hazard ratios (HRs) calculated, including an interaction term between volume and frequency. The interaction was confirmed with Altman and Bland’s method (results not presented) [27]. Covariates were selected for the multivariable model at *p* < 0.2 in the univariable model, and dropped if correlated with another covariate (at ρ > 0.5 and *p* < 0.05). The final multivariate model was built using manual stepwise backward removal of covariates at *p* < 0.05 [27]. The proportional hazards assumption was checked.

## Results

### Feasibility outcomes

**Fig. 1:**
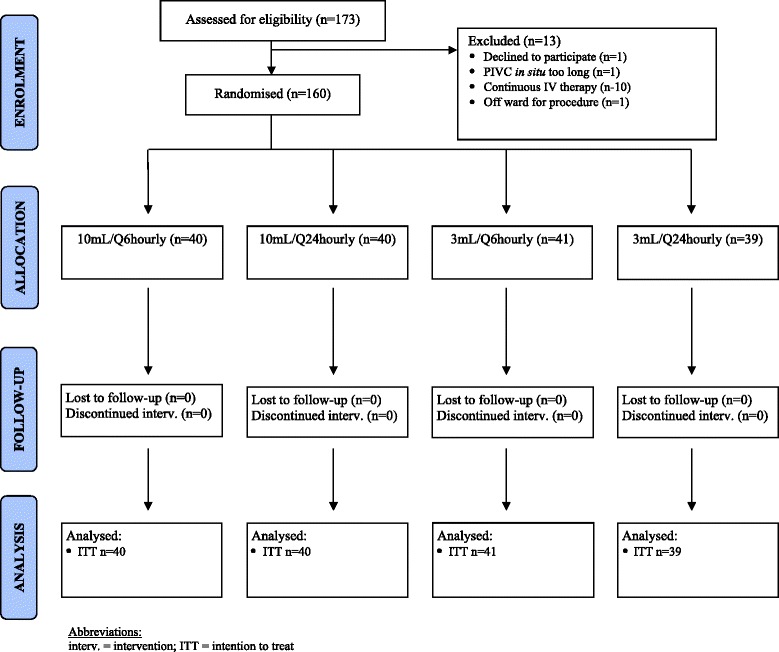
Participant flowchart

One hundred and seventy-three patients were screened for eligibility, with 160 recruited (Fig. 1). One hundred percent of recruited patients were randomised, giving an average of 40 patients per month. Staff on study wards were cooperative and supportive of the trial. RNs spent an average of 4 hours a day (Monday to Friday) recruiting and data collecting for the trial. Only follow-up data were collected on weekends for approximately 2 hours a day when RNs covered for a range of studies. All participants received the allocated intervention with no protocol violations, and no patients were lost to follow-up.

### Patients and PIVCs

At baseline, groups were similar in demographic and clinical risk profiles (Table 1). PIVCs were used for an average 3.1 (median: 2.70) days across all study groups and total 11,911 PIVC hours were studied (Table 2). The overall PIVC failure rate was 46 % or 6.13/1000 device hours, with occlusion and phlebitis the most common reasons for PIVC failure. No confirmed PIVC-related infections occurred in any group. Two suspected PIVC infections occurred; one patient had gram-negative bacilli in both peripheral blood and urine, the second had a blood culture with probable skin contaminant.

**Table 1:** Patient and insertion characteristics at baseline (*n* = 160)

Characteristic	Total (*n* = 160)	Flush volume		Flush frequency	
		3 mL (*n* = 80)	10 mL (*n* = 80)	24 h (*n* = 79)	6 h (*n* = 81)
Group size	160 (100)	80 (50)	80 (50)	79 (49)	81 (51)
Age (median, IQR)	63 (24)	63 (22)	64 (30)	63 (26)	64 (23)
Gender (male)	93 (58)	53 (66)	40 (50)	47 (59)	46 (57)
Overweight/obese^a^	70 (44)	33 (41)	37 (46)	33 (42)	37 (46)
Comorbidities (≥2)	110 (69)	51 (64)	59 (74)	57 (72)	53 (65)
Skin integrity (poor)	24 (15)	12 (15)	12 (15)	12 (15)	12 (15)
Infection (any type)	43 (27)	22 (28)	21 (26)	18 (23)	25 (31)
Antibiotic treatment	115 (72)	57 (71)	58 (72)	60 (76)	55 (68)
Frequency of IV tx^b^:					
- 0	15 (11)	10 (12)	5 (6)	8 (10)	7 (9)
- 1–2 times daily	23 (17)	10 (12)	13 (16)	14 (18)	9 (11)
- 3–4 times daily	71 (54)	35 (44)	36 (45)	37 (47)	34 (42)
- ≥5 times daily	23 (17)	13 (16)	10 (12)	8 (10)	15 (19)
Inserted in dominant arm	80 (52)	37 (47)	36 (49)	36 (48)	37 (47)
Insertion point:					
- post. wrist	39 (24)	22 (28)	17 (21)	18 (23)	21 (26)
- hand	38 (24)	18 (22)	20 (25)	18 (23)	20 (25)
- post. u/l forearm	30 (19)	15 (19)	15 (19)	17 (22)	13 (16)
- ant. u/l forearm	29 (18)	15 (19)	14 (18)	14 (18)	15 (19)
- cub. fossa/ant. u arm	24 (15)	10 (12)	14 (18)	12 (15)	12 (15)
Vein quality (poor)	85 (53)	41 (51)	44 (55)	39 (49)	46 (57)
Insertion in ward^c^	118 (74)	59 (74)	59 (74)	59 (75)	59 (73)
Inserted by:					
- IV nurse inserter	72 (45)	36 (45)	36 (45)	39 (49)	33 (41)
- any doctor	68 (43)	35 (44)	33 (41)	35 (44)	33 (41)
- any nurse/unknown	20 (13)	9 (11)	11 (14)	5 (6)	15 (19)
Multiple insertion attempts	32 (20)	15 (19)	17 (22)	17 (22)	15 (19)
Device size^d^:					
- 22 gauge	76 (48)	37 (46)	39 (49)	35 (44)	41 (51)
- 20 gauge	67 (42)	33 (41)	34 (42)	35 (44)	32 (40)
Extension tubing	82 (51)	41 (51)	41 (51)	43 (54)	39 (48)
3-way tap	118 (74)	59 (74)	59 (74)	59 (75)	59 (73)

*n* (%) shown unless otherwise indicated *post.* posterior, *ant.* anterior, *cub.* cubital, *mL* millilitres, *h* hours, *tx* treatment, *u*upper, *l* lower

^a^ Estimated

^b^ Frequencies may not add up to group size due to missing/omitted values

^c^ Versus all other

^d^ Other categories omitted

**Table 2:** Device outcomes at removal

Outcome	Total (*n* = 160)	Flush volume		Flush frequency	
		3 mL (*n* = 80)	10 mL (*n* = 80)	24 h (*n* = 79)	6 h (*n* = 81)
Device failure:	73 (46)	29 (36)	44 (55)	32 (41)	41 (51)
- occlusion or leaking	22 (14)	12 (15)	10 (12)	9 (11)	13 (16)
- infiltration	18 (11)	4 (5)	14 (18)	10 (13)	8 (10)
- phlebitis or too painful	22 (14)	7 (9)	15 (19)	8 (10)	14 (17)
- dislodgment	15 (9)	5 (6)	10 (12)	7 (9)	8 (10)
Device dwell time (days)^a^	2.70 (1.66–4.02)	2.23 (1.46–4.32)	2.73 (1.71–3.98)	2.83 (1.65–4.25)	2.26 (1.67–3.58)
Device-hours	11,911	5,998	5,914	6,327	5,584
Incidence rate (95 % CI)^b^	6.13 (4.87–7.71)	4.84 (3.36–6.96)	7.44 (5.54–10.0)	5.06 (3.58–7.15)	7.34 (5.41–9.97)
Log-rank test (*p* value)	-	0.063		0.054	

*n* (%) shown unless otherwise noted

*CI* confidence interval

^a^ Median and inter-quartile range

^b^ Per 1000 device-hours

### Primary endpoint

**Fig. 2:**
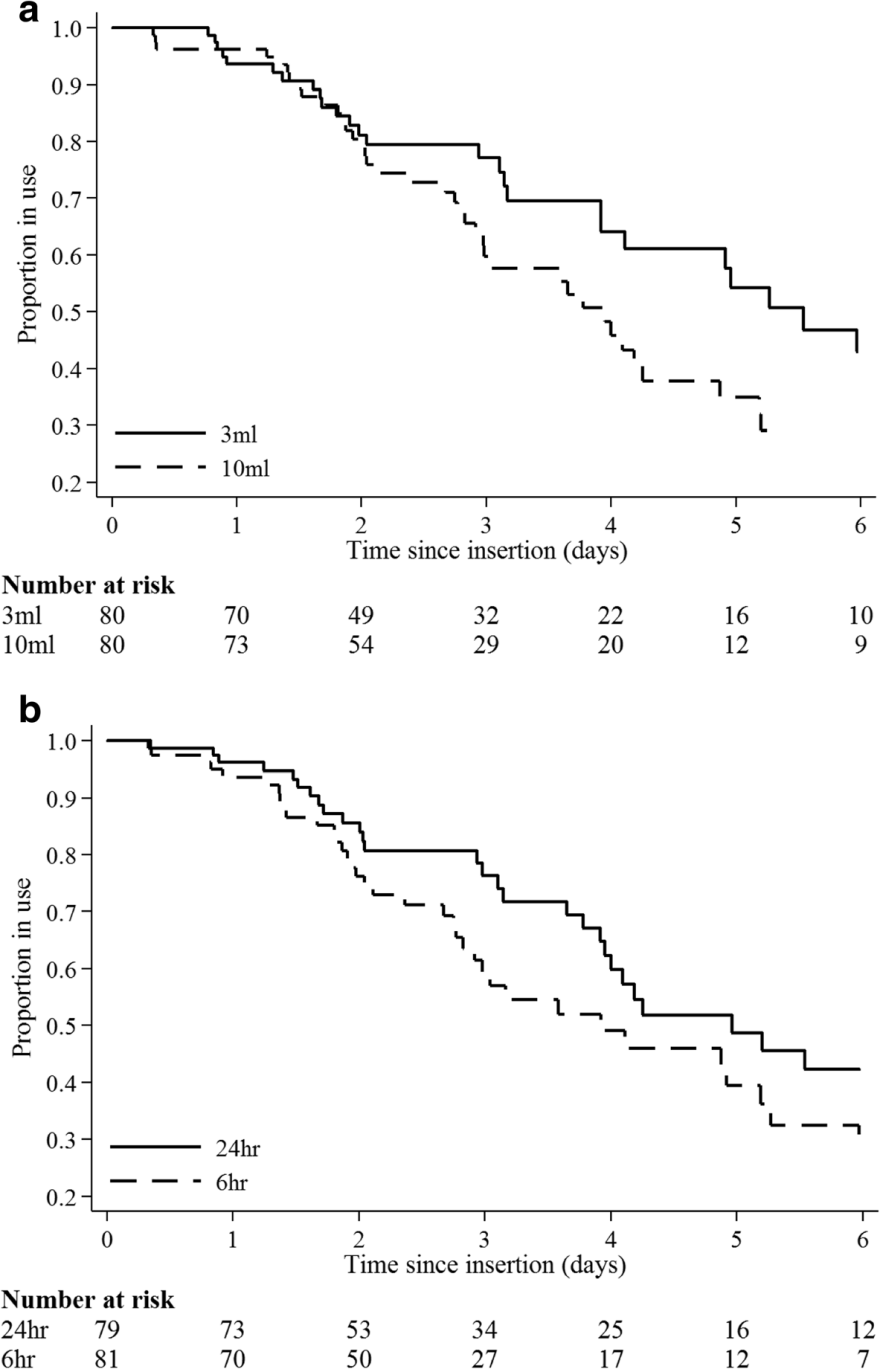
Kaplan-Meier survival from PIVC failure (*n* = 160) by **a** flushing volume (*p* = 0.063, log-rank) and **b** flushing frequency (*p* = 0.054, log-rank)

The incidence rate of PIVC failure in patients assigned to 3 mL flushing was 4.8 per 1000 hours compared with 7.4 per 1000 hours in those assigned to 10 mL flushing (*p* = 0.063, log-rank, Table 2 and Fig. 2). The incidence rate of PIVC failure in patients assigned to 24 hour flushing was 5.1 per 1000 hours compared with 7.3 per 1000 hours in those assigned to 6 hour flushing (*p* = 0.054, log-rank, Table 2 and Fig. 2). There was no significant interaction between the two study interventions (*p* = 0.21, Cox, Table 3); i.e. the results for comparison of the two volumes were not affected by the frequency, and the results for comparison between the two frequencies were also not affected by the volume. Univariable Cox regression found estimates of effect for both the volume and frequency interventions to be large but not statistically significant (Table 3). Failure was increased with 10 mL (versus 3 mL) volume (HR 1.56, 95 % CI 0.97–2.50, *p* = 0.06), and 6 hour (versus 24 hour) flushing (HR 1.58, 95 % CI 0.99–2.53, *p* = 0.06). On multivariable analysis, volume, frequency and their interaction remained non-significant, but female gender (HR 2.2, 95 % CI 1.3–3.6, *p* < 0.01), insertion in hand/posterior wrist (HR 1.7, 95 % CI 1.0–2.7, *p* < 0.05) and increased episodes rate per day (combined flushes and medication pushes) (HR 1.2, 95 % CI 1.1–1.4, *p* < 0.01) significantly predicted PIVC failure. Increasing the average daily episode rate by one (e.g. from three to four episodes per day) was associated with a 25 % increase in the relative risk of failure.

**Table 3:** Cox proportional hazards regression (*n* = 160)

	Univariable	Multivariable
	HR (95 % CI)	HR (95 % CI)
10 mL volume (referent: 3 mL)	1.56 (0.97–2.50)*	^f^
6 hr frequency (referent = 24 h)	1.58 (0.99–2.53)*	^f^
Interaction of volume and frequency^a^	0.54 (0.21–1.42)	^f^
Age (one year increase)	1.00 (0.99,1.02)	^f^
Gender (female)	1.90 (1.18,3.05)***	2.17 (1.33,3.55)***
Weight (over/obese)	1.29 (0.81,2.05)	^f^
Comorbidities (one category higher)^b^	1.03 (0.78,1.35)	^f^
Insertion on dominant side	1.67 (1.01,2.76)**	^f^
Infection at baseline (any)	1.56 (0.93,2.59)*	^f^
Antibiotics at baseline (yes)	0.99 (0.60,1.64)	^f^
IV treatment at baseline (yes)	0.95 (0.43,2.07)	^f^
Insertion at hand/posterior wrist^c^	1.67 (1.04,2.70)**	1.66 (1.02,2.69)**
Insertion at forearm^a^	0.58 (0.35,0.97)**	^e^
Insertion at cub. fossa/ant. upper arm^c^	1.01 (0.53,1.93)	^f^
Inserted by IV service^c^	0.95 (0.59,1.52)	^f^
Inserted by nurse^c^	2.01 (1.11,3.63)**	^f^
Size (20 g versus 22 g)	1.14 (0.70,1.87)	^f^
Vein quality (poor)	1.26 (0.79,2.01)	^f^
Multiple insertion attempts (yes)	1.32 (0.78,2.23)	^f^
Extension tubing (yes)	1.25 (0.78,1.98)	^f^
3-way tap (yes)	1.33 (0.79,2.24)	^f^
IV treatment at device removal (yes)	2.61 (1.28,5.29)***	^e^
IV treatment rate at device removal^d^	1.40 (1.07,1.82)**	^e^
IV antibiotics at device removal (yes)	2.07 (1.27,3.38)***	^e^
IV antibiotics rate at device removal^d^	1.33 (1.09,1.62)***	^e^
Medication types administered:		
- antibiotics^c^	1.12 (0.67,1.87)	^f^
- cephalosporins^c^	0.80 (0.49,1.32)	^f^
- penicillin combination^c^	0.75 (0.40,1.41)	^f^
Episode rate (medications and flushes)	1.22 (1.08,1.38)***	1.25 (1.10,1.41)***

*HR* hazard ratio, *CI* confidence interval, *IV* intravenous

*p value <0.2; **p value <0.05; ***p value <0.01

^a^Model for the interaction term results included the main effects

^b^Ordinal: 0 = none/one, 1 = two/three, 2 = four or more

^c^Versus all others

^d^Per day, ordinal: 0 = none, 1 = one/two, 2 = three/four, 3 = five or more

^e^Excluded due to correlation with other covariate

^f^Covariate not entered into multivariate model (at *p* ≥ 0.2), or removed during model building at *p* > 0.05. Only variables *p* < 0.2 on univariable analysis entered into the multivariable model

## Discussion

This pilot study was initiated in response to an unacceptably high PIVC failure rate in our organisation and resultant high morbidity and costs [10, 12]. This is one of the first RCTs to evaluate the impact of different flushing volumes and frequencies on PIVC outcome. Feasibility of a future large trial was indicated, with the interventions acceptable to ward staff and feasible to conduct in the clinical setting as demonstrated by the protocol adherence and successful recruitment rate. Initial group comparisons suggested that higher frequency and higher volume flushing were associated with increased PIVC failure. This is counter-intuitive to current practice, where it is believed more often and larger volume flushing equals less PIVC failure. However, univariable and multivariable regression showed that flushing volume and frequency were not significantly associated with failure. Retrospectively, the study power was 40 % and 14 % for the volume and frequency hypotheses respectively; larger, definitive trials would require 140 patients/group (volume), and 520 patients/group (frequency) (90 % power, alpha 0.05, https://stat.ubc.ca/~rollin/stats/ssize/).

Similar to our results, a single-site trial (*n* = 397) of different flushing frequencies (3 mL twice daily versus 3 mL once daily) in Italy also found no significant difference in risk of PIVC failure (12.1 % versus 9.5 % *p* = 0.42) [22]. However, their sample was a select paediatric population (receiving no infusion therapy or intravenous antibiotics, and using prefilled flush syringes), quite different from our cohort. Additionally, their observed overall proportion of failure (8.7 %) was very low compared to other studies. This suggests that the use of prefilled and/or pressure limiting syringe technology needs to be explored to optimise flush and medication administration.

PIVC failure was significantly associated with the increasing episode rate of PIVC accesses per day. However, medication type (i.e. drug or antibiotic classification) was not a significant contributing factor to PIVC failure. So if it is not the flush volume, frequency or medication type that predicts failure, it may be the excessive injection pressure associated with manually prepared syringes that damages the vessel intima, by direct pressure and/or haemodilution activating the endothelium [17]. It could also be the manual handling of the PIVC during administration.

Our recent survey of flushing practices (*n* = 1178) revealed 23 % of nurses/midwives used a 5 mL or 2 mL syringe to deliver a flush, and almost all used manually prepared flush syringes [20]. This implies a lack of appreciation for the increase in pressure per square inch associated with the properties of smaller size syringes. The use of reduced pressure for flush delivery through a syringe with a larger diameter such as the standard 10 mL syringe is recommended to optimise flush outcomes and minimise damage to the vein [17, 28, 29]. These recommendations are largely derived from physics principles, and have not been tested in clinical trials. In recent times, commercially prepared prefilled flush syringes have become available, which negate the potential for operators to make an incorrect choice of a smaller size syringe, since they are produced in diameters consistent with a 10 mL syringe, but in 3, 5 and 10 mL volumes.

Female gender was significantly associated with PIVC failure (HR 2.2), consistent with a previous study where females were significantly more at risk for both phlebitis (HR 1.64, *p* < 0.001) and occlusion (HR 1.44, *p* < 0.001) [30]. While gender is a non-modifiable factor, staff should take this high risk factor into consideration for best-practice insertion, monitoring and maintenance regimens.

We also identified hand/wrist insertion as a significant risk for PIVC failure (HR 1.7). Previous research confirmed insertion site as a predictor of PIVC failure. Again, insertion in the hand compared to the forearm had an HR of 1.47 (*p* < 0.001) for occlusion and an HR of 2.45 (*p* < 0.001) for accidental removal in previous work [30]. Other research has linked insertion site with phlebitis [14, 31, 32]. PIVC insertion site is a variable over which clinicians can exercise judgement and choice, and they should therefore consider these risks in relation to their insertion practice. Guideline site recommendations have recently been updated to preference the forearm [18, 24, 33].

It is clear that the mechanisms of how IV flushing, medication and fluid administration impact on PIVC complications require further elucidation. The literature to date, including Schreiber and colleagues (2015) [22] and this pilot study, have not identified effective flushing regimens. There are few RCTs performed on PIVC flushing, and this study provides new, rigorous data that contribute to knowledge in this area. More recently, clinically indicated replacement has allowed catheters to be used for longer periods, so trials such as this are needed to investigate how improved maintenance can keep PIVCs functional over time [34].

### Limitations

The small pilot sample and single site setting impact on the interpretation and generalisability of the results. It was not possible to mask the respective interventions, and so there was potential for outcome assessment bias. The use of dedicated research nurses, standardised data collection and blinded microbiologists minimised the risk. We did not control flushing volumes used pre- and post-medications, and this may have confounded the effect of the randomised flushing. However, analysing the number of total PIVC accesses per day appears to have controlled for this factor. The recommended syringe size for all flushes was a 10 mL syringe; however, we do not know if this was always used, and excessive pressure may have been a confounder if smaller sizes were used.

## Conclusions and implications

Larger, definitive trials of flushing volume and flushing frequency of peripheral intravenous catheter management are feasible and required. Neither increased flushing volume nor frequency significantly altered the risk of PIVC failure in this pilot trial; however, the trial had inadequate power. (Female gender, hand/posterior wrist placement and frequency of access (flushes and medication) should be considered confounders in future trials.) The mechanisms of how IV flushing, medication and fluid administration impact on the cannula, vessel endothelium and blood components are poorly understood and require further explication. We need to explore how syringe technology and method of administration can make a difference to PIVC outcomes and therefore transform the delivery and patient experience of IV care.

## Abbreviations

CI, confidence interval; HR, hazard ratio; IV, intravenous; PIVC, peripheral intravenous catheter; RCT, randomised controlled trial; RN, Research Nurse
